# Effect of SARS-CoV2 S protein on red blood cells parameters. Some comments

**DOI:** 10.1016/j.bj.2025.100857

**Published:** 2025-04-22

**Authors:** Geir Bjørklund, Umberto Tirelli, Salvatore Chirumbolo, Luigi Valdenassi

**Affiliations:** a Council for Nutritional and Environmental Medicine, Mo i Rana, Norway; b Tirelli Medical Group, Pordenone, Italy; c Department of Engineering for Innovation Medicine, University of Verona, Verona, Italy; d Department of Internal Medicine, University of Pavia, Pavia, Italy

Dear Editor,

Dima et al., reported that SARS-CoV2 Spike proteins are able to affect erythrocyte biology [1]. Previous reports showed that SARS-CoV2 spike proteins cause haemagglutination, probably due to the well-known role of glycoconjugates on red blood cells membranes [2]. The authors did not specify the molecular hallmarks of their used Spike proteins (ACRO Biosystems; Newark, DE, USA) [1]. Which kind of Spike protein product they selected? For example, for the Omicron variant, the authors never specified if the subvariant BA.4, or XBB or BA 2.75 or BA 2 + L452 M or so forth. The genericity of their methodological description asks for a rebuttal from theirs.

They referred to a previous paper for doses [3]. However, in Craddok et al.’s paper, the majority of plasma Spike protein concentration is below 2 ng/ml in acute infected COVID-19 patients, as 20 ng/ml is an extremely rare event [3]. The activity of 20 ng/ml might be an unspecific effect of the trimeric Spike protein in agglutinating cells and/or modifying erythrocytes membranes, particularly if controls did not assess this question.

Alpha and Delta variants increased MCV both at 2 and 20 ng/ml final concentrations and that RDW increased significantly if erythrocytes were challenged with Omicron recombinant Spike protein [1]. In this respect, control selection is particularly concerning.

While the authors used only physiological saline as controls, they did not introduce a control for the unspecific reaction of proteins. This is strategically important, as they tailored their paper on the different Spike protein variants [1]. Furthermore, to confirm that the assay is functioning correctly, a positive control (such as a protein or antibody known to agglutinate red blood cells) should be included. This ensures that the assay system is capable of detecting true agglutination.

SARS-CoV2 Spike proteins, simply binding erythrocytes. induce impaired MPV and consequently RDW. In a recent paper by Grobbelaar et al. the authors demonstrated that, challenging whole blood with simply 1 ng/ml SARS-CoV2 Spike protein, proteins induce red blood cells agglutination, despite the very low dose of Spike molecules, whereas untreated whole blood showed regularly shaped (normocytic) erythrocytes [4].

I think it could be obvious, for a clinical biochemist, to envisage an impaired MPV and RDW parameters if microagglutination occurs in testing vials.

## Declaration of competing interest

The authors state they have no conflict of interest.
